# Vaccine Adverse Events Reported during the First Ten Years (1998–2008) after Introduction in the State of Rondonia, Brazil

**DOI:** 10.1155/2013/853083

**Published:** 2013-02-14

**Authors:** Mônica P. L. Cunha, José G. Dórea, Rejane C. Marques, Renata S. Leão

**Affiliations:** ^1^Department of Nursing, Fundação Universidade Federal de Rondônia, 76801-974 Porto Velho, RO, Brazil; ^2^Faculty of Health Sciences, Universidade de Brasília, 70919-970 Brasilia, DF, Brazil; ^3^Universidade Federal do Rio de Janeiro, Campus Macaé, 27971-550 Rio de Janeiro, RJ, Brazil; ^4^Instituto de Biofísica Carlos Chagas Filho, Universidade Federal do Rio de Janeiro, 21941-902 Rio de Janeiro, RJ, Brazil

## Abstract

Despite good safety records, vaccines given to young children can cause adverse events. We investigated the reported adverse events following immunization (AEFI) of vaccines given to children of less than seven years of age during the first ten years (1998 to 2008) in the state of Rondonia, Brazil. We worked with the events related to BCG (Bacillus Calmett-Guérin), HB (hepatitis B), DTwP/Hib (diphtheria-tetanus-pertussis+Hemophillus influenza b), DTP (diphtheria-tetanus-pertussis), MMR (mumps, measles, rubella), and YF (yellow fever) vaccines because they were part of the recommended scheme. The number of doses of vaccines given was 3,231,567 with an average of AEFI of 57.2/year during the studied period. DTwP/Hib was responsible for 298 (57.8%), DTP 114 (22.9%), HB 31 (6%), MMR 28 (5.4%), BCG 24 (4.7%), and YF 20 (3.9%) of the reported AEFI. The combination of the AEFI for DTwP/Hib vaccines showed the highest number of systemic (61.4%) and local events (33.8%). Young children (≤1-year old) were more susceptible to AEFI occurring in the 6 hours (54.2%) following vaccine uptake. This study suggests significant differences in reactogenicity of vaccines and that despite limitations of the AEFI Brazilian registry system we cannot ignore underreporting and should use the system to expand our understanding of adverse events and effects.

## 1. Introduction

 Immunization is an essential component of public health policies to control infectious diseases. Its success in worldwide eradication of smallpox and, regionally, in clearing out other infectious diseases makes vaccines one of the most trusted cost-effective public-health interventions [1]. Therefore, because of its universal use which in many cases or circumstances is mandatory, issues of effectiveness and safety take on paramount importance. Despite the stringent protocol for vaccine licensure, some individuals can react to the vaccine's antigens (and formulation ingredients). Discomfort, induration at the site of the inoculation, and pain are common features following vaccination in children but are regarded as inconsequential. However, more serious events, albeit rare, can occur in susceptible individuals [2]. 

 As vaccine coverage increases for the intended infectious diseases, and additional recommended booster doses, there has been an attendant increase in the number of doses a child is now receiving. Therefore, as a consequence of the increased number of vaccinations the risk of an adverse event is proportionally increased. Paradoxically, when the immunization program is effective the incidence of vaccine-preventable disease will drop, resulting in higher numbers of adverse events following immunization (AEFI) and its easier perception [3]. Indeed, in the USA, the number of reported AEFI (registered by the vaccine adverse event reporting system-VAERS) exceeded the incidence of most preventable childhood diseases combined [3]. 

 Vaccine safety surveillance and followup are central to address actual and perceived issues related to AEFI [4]. However, such surveillance and followup infrastructure lags behind vaccine development in industrialized countries [3] and is absent in most developing countries. As an example, despite the known toxicity of mercury, until the early 2000s there was no research on the toxicology of low doses of Thimerosal/ethylmercury used as preservative in certain vaccines [1]. As a result, a significant gap between perceived and actual risks has developed, and now we need appropriate strategies to maintain the high vaccination rates which are crucial to control infectious diseases [5]. Parents' trust, in tandem with maintaining a high uptake of vaccines, relies mainly on primary health care workers.

 Because pharmaceutical products carry risks, vaccines are no exception; however, expected serious events are rare; furthermore, prior to its licensure, as observed by Autret-Leca et al. [6] the size of clinical trials is insufficient to identify rare or deferred adverse effects. Therefore, AEFI systems are important tools to monitor temporal associations between vaccination and a suspected serious or mild adverse or unintended reaction to a given licensed product. The information collected can be used by government agencies in charge of public health, stakeholders, vaccine manufactures, scientists, and the general public. Zhou et al. [7] summarized the objectives of such systems as “(1) detect new, unusual, or rare vaccine adverse events; (2) monitor increases in known adverse events; (3) determine patient risk factors for particular types of adverse events; (4) identify vaccine lots with increased numbers or types of reported adverse events; and (5) assess the safety of newly licensed vaccines.” As a consequence, such systems will be central to give public health authorities the necessary plasticity to act rapidly and accurately on an unintended effect of a specific vaccine. 

 Most AEFI systems are passive and designed for the specific purpose of pharmacovigilance of vaccines; therefore, beyond that, they are of limited use. Nevertheless, they all share a central role in providing crucial information regarding safety of postlicensure vaccine monitoring [8]. Inherent limitations related to diversity of vaccine type, vaccination schemes, and adopted protocol of AEFI by countries, as well as vaccine brands, have made it difficult to compare outcomes. Therefore it is crucial to understand and use the existing AEFI systems in order to improve competence and build expertise among public health workers in dealing with the uncertainties that surround vaccine-related unintended effects.

 Since 1998, Brazil has implemented a nationwide program of reporting AEFI. The objective of this work was to use the notified adverse events in the state of Rondônia during the first ten years of the program's implementation as a case study to address current safety issues related to vaccines used in children.

## 2. Materials and Methods

 The study was approved by the Ethics Committee of the Universidade Federal de Rondônia (protocol #43/09). AEFI data (*Eventos Adversos Pos Vacina-EAPV*) were collected directly from the specific pharmacovigilance agency (*Gerência do Programa de Imunizações da Secretaria Estadual de Saúde de Rondônia*). The data are presented collectively in order to safeguard the integrity and anonymity of those involved (patients and health care agents), and results are used only for the purpose of the study and not as an advocacy for or against a specific vaccine. 

 Vaccines in Brazil are free of charge to the consumer and are available in all public-run (federal, state, or municipal) hospitals, or equally public-run medical clinics throughout the country. In large cities, vaccines are also available from the private clinics where Thimerosal-free and brand-name products can be purchased. Most vaccines in newborns and young children are delivered in primary care vaccine clinics run by local public health qualified professionals; public health nurses or specifically trained health professionals administer the vaccinations and provide guidance for parents. In the maternity wards of public institutions, vaccination of newborns against hepatitis B is mandatory in the first 24 hours after birth; for mothers delivering in private institutions, this vaccine is procured later in private and public medical centers. 

 Adverse events here are defined “as any severe and/or unexpected adverse sign or symptom occurring after vaccination” [9]. A detailed account of how the Brazilian AEFI system is organized is described by Waldman et al. [9]. The data is captured by primary care medical offices and hospitals. Public health professionals (doctors and nurses specifically in charge of postvaccination adverse events) are required to report postvaccine events that received medical attention. All information is compiled in a specific form that is digitalized and electronically sent to a central office (*Programa Nacional de Imunização-PNI*). The notification form is a structured sheet that captures data related to the patients (age, sex, date of vaccine, and the adverse event); the characteristic of the adverse event is provided and encompasses systemic and localized reactions. The occurrence of an AEFI in a hospital or in medical clinics is required to be reported to the specific state or regional office; the first stage of data generation is by attending physician that reports on the evolution and releases the structured form to the AEFI office. However, after the registry, there are no verification checks. Once the information is properly entered in the appropriate form (with a structured list of 48 AEFI items) it is digitalized into the national data bank. Public medical offices (*postos de saúde*), where most vaccines are dispensed, are rarely contacted for adverse events. However, the processing office is part of all state-run hospitals; private-run vaccination clinics and hospitals are required to report AEFI in the same format. 

 The Brazilian reporting system of adverse events started in 1998 in a systematic form as specified in the “*Manual de Vigilância Epidemiológica dos Eventos Adversos Pós-Vacinação.*” In year 2000 a national program of vigilance of adverse events was implemented for the entire country (*Sistema de Informação da Vigilância Eventos Adversos Pós-Vacinação, SI-EAPV*). Although the system is backed by the national health authority there is no legal provision to compensate or attenuate sequels, other than what is provided by state run hospital and services.

 Since then the SI-EAPV information on AEFI has been provided in a consistent and regular fashion by all states. We included all notified cases of adverse events attributed to any of the vaccines during the studied period (2000 to 2008) for children less than seven years of age. The years 1998 and 1999 were not available in the agency database. We excluded all cases with duplicated notification (only five cases) and those with incomplete information, that is, those cases without date of vaccine administration, type of vaccine, and associated AEFI symptoms (only six cases). Localized reactions that required hospitalization for more than 24 hours were counted as systemic. We considered the following vaccines: BCG (Bacillus Calmett-Guérin), HB (hepatitis B), DTP+HIB (diphtheria-tetanus-pertussis+Hemophillus influenza b), DTP (diphtheria-tetanus-pertussis), MMR (mumps, measles, rubella), and YF (yellow fever); they represented 516 of a total of 530 cases in the period.

 Data were summarized with the Statistical Package for Social Sciences(SPSS) version 12.0 (IBM Corporation,Somers, NY, USA) and Microsoft Office EXCEL software (version 2007; Microsoft Corp, Redmond, WA, USA). Statistical analysis with Friedman and Kendall test was applied with software *Statistica *7.0 (Tulsa, OK, USA). 

## 3. Results

**Figure 1:**
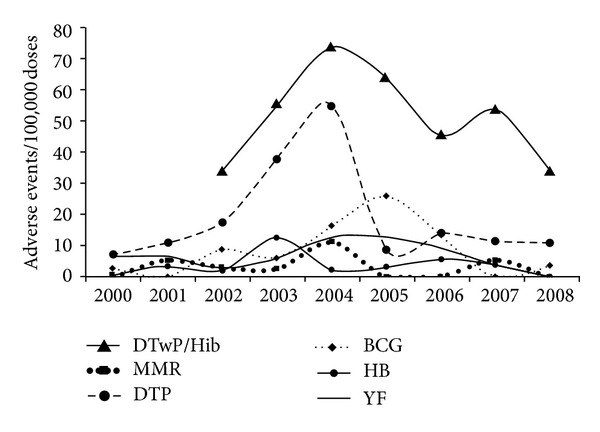
Distribution of notified adverse events and vacines; Rondônia 2000−2008. Source: “*SI-EAPV do Programa de Imunizações do Estado de Rondônia*” (EAPV Registry of Rondonia). Statistically significant differences between vaccines (Friedman ANOVA and Kendall Coefficient of concordance; ANOVA Chi Square (*N* = 8, df = 6) = 34.48227; *P* = 0.00001 Coefficient of concordance = 0.71838). BCG: Bacillus Calmett-Guérin; HB: hepatitis B; DTP+Hib: diphtheria-tetanus-pertussis+Hemophillus influenza b; DTP: diphtheria-tetanus-pertussis; MMR: mumps, measles, rubella; YF: yellow fever.

 The rate of adverse events per individual vaccine is shown in Table 1 and is also illustrated in Figure 1. The time series illustrates a different pattern for the DTP and DTwP/Hib vaccines. These vaccines showed the highest rates of AEFI (52.7 and 70.6 per 100,000 resp.,). Year 2004 was particularly high only for these vaccines with the tetravalent (DTP/DTwP/Hib) maintaining the highest levels during the study; coincidently this year had the highest number of vaccinated children. Indeed of the 516 total cases for all vaccines, the tetravalent accounted for more than half (298). The other vaccines (HB, MMR, BCG, YF) together showed less than 20%.

**Figure 2:**
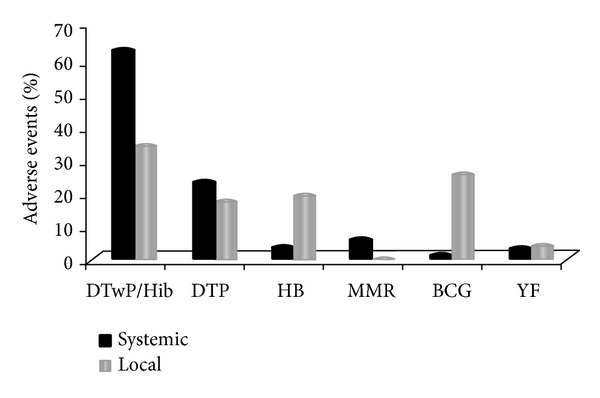
Type of reported adverse event and type of vaccine; Rondônia 2000–2008. Source:** “** *SI-EAPV do Programa de Imunizações do Estado de Rondônia*” (EAPV Registry of Rondonia). BCG: Bacillus Calmett-Guérin; HB: hepatitis B; DTP+Hib: diphtheria-tetanus-pertussis+Hemophillus influenza b; DTP: diphtheria-tetanus-pertussis; MMR: mumps, measles, rubella; YF: yellow fever.

 The distribution of adverse events as a function of the type of the reaction—systemic or local—is shown in Figure 2. Overall, systemic adverse events accounted for 86.6% of all the reported cases and the tetravalent vaccine (DTwP/Hib) showed the highest rate of systemic (61.4%) as well as local (33.8%) events. For the adverse events considered unexpected or of serious gravity, the DTP and Hib were reported in 57.1% of the cases, and HB alone was responsible for 21.4%. Together, the DTwP/Hib/DTP vaccines showed the highest rate for both systemic and local reactions; as for the other vaccines, the pattern was different for local (BCG>HB>YF>MMR) and systemic (MMR, HB>YF>BCG) reactions.

 The most frequent systemic adverse events were hypotonic-hyporesponsive episodes (HHEs), fever >39.5°C, febrile convulsion, and generalized exanthema. HHE was almost exclusively related to the DTP (30.4%) and DTwP/Hib (41.6%) vaccines. These vaccines were also related to 75% of convulsions without fever, and 69.5% of fever (≤39°C), as well as local reactions with reddening, pain, and induration. Most of the EAPI (54.2) were reported to occur in <6 hs after the vaccine in children <12 months. 

 It is notable that in the majority of cases the AEFI occurred after the first and second dose of the vaccine and more frequently in children of less than 12 months of age. In more severe cases, like HHE, the recommendation is that the following vaccination should be conducted in a hospital facility; in cases of fever and seizures, the regular use of analgesic and antipyretic medication is advised. 

## 4. Discussion

 The notified AEFI seem to be associated more with the type of vaccine than with the child's age. The first vaccine (HB) is given on the first postnatal day, and this showed the second highest rate of AEFI, but the vaccine that produced the highest rate of AEFI (DTP/DTwP/Hib) is usually administered at two months of age. It is important to emphasize that in Brazil the rate of reported AEFI is positively associated with the human development index of the state [10], suggesting that underreporting occurs. Indeed, Gomes Monteiro et al. [10] showed the lowest reported AEFI of DTP (from 2002 to 2005) to be in the Northern region of Brazil which has the lowest development indices in the country. Therefore, it is likely that the notified AEFI are underestimated for the state of Rondonia, a representative Northern state.

 Fever, HHE, and seizures were the most common systemic AEFI reported in this study. Although in the case of DTwP/Hib (along with convulsions) recovery without after effects was achieved in 98.4% of the cases [11], practical counseling does exist, which can help improve management of cases. Fever, irritability, and feeding disturbances (even anorexia) are transient adverse events that are widely observed, especially in children aged less than seven months, and these events may impact breastfeeding rates and nutrition; in such cases it is important to remind parents that breastfeeding protects against decreased energy intakes, decreasing pain and alleviating discomfort and stress of vaccination in very young children [12].

 AEFI can vary as a function of the vaccine (formulation and manufacturers), vaccinee (age of children), and country (vaccination scheme adopted, AEFI reporting systems, and adverse event compensation policies); hence the difficulty in comparing outcomes of AEFI within and between countries. Because of such difficulties, this is a subject that has not been well studied [3], and we can only make inferences from indirect sources. Evans [13] compiled information on compensations available for a few countries and showed that compensated claims (which refer to severe AEFI) by vaccine type differ greatly. It seems that DTP was, at the time of the study, the vaccine showing the highest claims [13] and still is the vaccine with the highest occurrence of AEFI in young children. 

 For few individuals AEFI are severe, acute, with outcome clearly perceived as harmful. When a severe adverse event happens, an individual bears a significant burden for the greater good or “herd immunity” [14]. Concerns with the long-lasting after effects of disabling illness caused by or associated with the use of vaccines have led a number of countries to create effective systems of surveillance for AEFI and respective compensation programs [14].

 During the last decade, however, we have seen an increased awareness related to AEFI that has extended beyond specific vaccine antigens to include low doses of excipients (preservative-Thimerosal and adjuvant-aluminum). Both ethylmercury (a breakdown product of Thimerosal) and aluminum are known neurotoxicants *per se* and, in the case of Hg, with a long history of known toxicity that also includes its organic form—ethylmercury [15]. These excipients (with neuronal effects) are used at low doses as part of some vaccine formulations (Thimerosal-containing vaccines [TCV], and aluminum-adjuvanted vaccines) and are considered safe. Indeed none of the rare neurologic adverse events (encephalopathy, Guillain-Barre syndrome, meningo-encephalitis, polyneuropathy, peripheral neuritis, *per se* or in combination) associated with vaccine-antigens [16] can be attributed to low doses of either mercury or aluminum. However, the untested concept of low-dose safety of these excipients originated in the 1930s in the wake of vaccine development. New experimental research designed to model low-dose exposure relevant to vaccines has established proof-of-concept that Thimerosal-Hg has the potential to produce nonclinical effects in the central nervous system [17] not contemplated by AEFI. 

 Since late 1990s industrialized countries of Europe and North America have restricted the use of Thimerosal as a preservative in vaccines intended for infants and young children. Although experimental studies can demonstrate toxic effects of low doses of Thimerosal we cannot predict neurological disorders for vaccine-Thimerosal. The few epidemiological studies taken together can at best be interpreted as inconclusive; they cannot show a clear association of ethylmercury with mental disability [18, 19]. However, several studies point to transient delays in neurodevelopment as measurable by neurobehavioral tests [20–23] as well as decreased pain associated with Thimerosal-free vaccine [24, 25]. Additionally, regarding immunologic effects, epidemiological studies have suggested an association of Thimerosal and patch-test sensitivity; countries that eliminated Thimerosal from vaccines such as Austria, Denmark, Poland, and the USA [26] observed a decrease in Thimerosal patch-test reactions. Moreover, both experimental [27] and clinical [28] studies have addressed autoimmune (autoinflammatory) syndrome induced by adjuvants (ASIA) in association with some adjuvanted vaccines. Although this emerging information is not part of the AEFI recording system the collected data are nevertheless shaping perceptions among health workers and stakeholders.

 The importance of the study is that it the first attempt to address the AEFI after its implementation. The main limitation is that this system in the state of Rondonia, one with a low score of developmental index, has no means to assess reporting bias (i.e., full medical histories of patients) or to estimate nonreported adverse events.

## 5. Concluding Remarks

There is a need to improve vigilance of vaccine adverse events and implement compensation for those few children that are severely affected by unintended effects of vaccines. Public health workers need to develop competence to interpret AEFI in this new era of increased infectious diseases prophylaxis by vaccination. Stakeholders should be served with the best and most reliable information to ensure that public health immunization policies can live up to their mission.

## Figures and Tables

**Table 1:** Distribution of reported adverse events and type of vaccine; Rondônia 1999–2008.

Event type	Vaccines											
	DTP		DTP/Hib		HB		MMR		YF		BCG	
	*N*	%	*N*	%	*N*	%	*N*	%	*N*	%	*N*	%
Systemic												
Headache and vomiting	3	2.61	6	2.01	0	0.00	1	3.57	0	0	0	0.00
Afebrile seizure	2	1.74	9	3.02	1	3.23	0	0.00	0	0	0	0.00
Febrile seizure	19	16.52	37	12.42	1	3.23	0	0.00	0	0	0	0.00
Induration	2	1.74	4	1.34	3	9.68	0	0.00	1	5	0	0.00
Hypotonic hyporesponsive episodes	35	30.43	124	41.61	1	3.23	1	3.57	0	0	0	0.00
Generalized rash	2	1.74	12	4.03	3	9.68	8	28.57	4	20	0	0.00
Fever ≥39.5°C	30	26.09	52	17.45	1	3.23	2	7.14	0	0	1	4.17
Fever ≤39.5°C	1	0.87	16	5.37	1	3.23	3	10.71	1	5	1	4.17
Other serious events and/or unusual	2	1.74	8	2.68	3	9.68	1	3.57	0	0	0	0.00
Hypersensitivity reaction to 2 h	2	1.74	3	1.01	1	3.23	5	17.86	5	25	0	0.00
Hypersensitivity reaction after 2 h	4	3.48	6	2.01	0	0.00	2	7.14	5	25	0	0.00
Others	1	0.88	2	0.67	0	0.00	2	7.1	2	10	0	0.00
Local												
Pain/redness/heat	4	3.48	7	2.35	0	0.00	0	0.00	1	5	0	0.00
Local hot abscess	4	3.48	2	0.67	13	41.94	1	3.57	0	0	0	0.00
Others	4	3.48	10	3.36	3	9.7	2	7.1	1	5	22	91.67

Total	115	100%	298	100%	31	100%	28	100%	20	100%	24	100%

Percentage (%) was calculated from the number of specific cases and total number of reported cases.

Source:** “** *SI-EAPV do Programa de Imunizações do Estado de Rondônia*” (EAPV Registry of Rondonia). BCG: Bacillus Calmette-Guérin; HB: hepatitis B; DTP+Hib: diphtheria-tetanus-pertussis+Hemophillus influenza b; DTP: diphtheria-tetanus-pertussis; MMR: mumps, measles, rubella; YF: yellow fever.
